# Severity in schizophrenia patients receiving atypical antipsychotic medications

**DOI:** 10.6026/973206300181154

**Published:** 2022-12-31

**Authors:** Syed Meraj Alam Fatmi1j, Rakesh Koul1, Prakash Suruchi, Sheenam Ayub

**Affiliations:** 1 Department of Pharmacology, KD Medical College, Hospital & Research Center, Mathura, India; 2 Department of Pediatric and Preventive Dentistry, KD Dental College & Hospital, Mathura

**Keywords:** Atypical antipsychotic drugs, Adverse drug reactions (ADR), GASS score, Risk factor, Probability

## Abstract

Atypical antipsychotic drugs are nowadays the mainstay of treatment of schizophrenia due to their lesser extrapyramidal symptoms (EPS) as adverse effects. However, these drugs have different profiles of adverse drug reactions (ADRs). Here, the objective of this study was to analyze the probability, occurrences, and more significant involvement of various risk factors. A prospective observational study was carried out on a patient with schizophrenia who has prescribed atypical antipsychotic drugs for their treatment. The probability of the ADR was analyzed by using the Naranjo causality assessment scale. While Glasgow antipsychotic Side effect Scale (GASS) was used to estimate the severity of side effects. Statistical software for social science (SPSS) ver 25; was used for different descriptive statistics and chi-square analysis. A total of 140 patients were included in the study of which the majority (58.57 %) was male. However, atypical antipsychotic drugs were primarily prescribed to the patient as mono therapy (81.43 %). Interestingly, COVID-19 infections were reported as positive in 39.29 % of total patients. Probability assessment of ADRs revealed that most (55 %) were "Probable". Subsequently, the GASS score was evaluated for severity, the majority (55.71 %) were reported as "Mild". The statistically significant association between gender and severity of side effects & duration of illness and severity of side effects were found (P>0.5).The Present study aids in knowing the risk factors and improving the management practices of ADR, thereby improving the guidelines in terms of safe clinical approaches for psychiatric patients.

## Background:

Adverse drug reactions (ADRs) are a global issue associated with the irrational usage of medications to treat different illnesses. ADRs are frequent and can interfere with patients' quality of life. In addition to the inherent risks associated with the medications themselves, specific and erratic drug sensitivity in each patient can result in ADRs. As a result, the prescribing practitioner needs to exercise substantial skill to choose and use the best and safest medications for a given individual from the wide range of options available [1].Beyond significantly influencing the patient's recovery, drug toxicity also has a detrimental effect on the healthcare industry's economy [2]. Since estimates of the cost of ADRs for hospitalized patients range from 2 to 4 billion dollars per year and for outpatient patients range from 30 to 136 billion dollars per year, the current economic crisis in healthcare necessitates a rigorous examination of ADRs as a means of reducing overall expenses [3].One of the most popular first-line treatments for schizophrenia and other psychotic disorders is an antipsychotic medication. Patients with bipolar disorder and other depressive disorders may also take these medications [4]. Antipsychotic drugs used to treat schizophrenia are frequently linked to some unpleasant side effects [5]. The main negative effects of antipsychotic medications include sedation, weight gain, sexual dysfunction, sleep difficulties, and alogia [6]. Extrapyramidal symptoms (EPS), metabolic, and cardiovascular adverse effects all constitute a serious threat to life and necessitate treatment [7]. Atypical antipsychotics agentswere believed to reduce both positive and negative symptoms of schizophrenia with a lower risk of EPS. These agents have been reported to produce different adverse effects like weight gain, seizures, insomnia, fatigue, etc. [8]. The inability to tolerate these adverse effects results in non-adherence and medication withdrawal, which might result in relapse. As a result of the relapse, the probability of job loss, bad social interactions, and suicide rose [9].The COVID-19 outbreak has caused people across the world to reevaluate standard medical care and services. The same has been applied to psychiatric therapies. In several nations,psychiatric facilities have been shut down or converted into COVID-19 wards, which has exacerbated the pressure on the psychiatric clinics still accepting patients and left many patients and their families with more unmet needs. Renowned professionals in the field have acknowledged the difficulties with COVID-19 for people with psychiatric conditions [10]. Therefore, it is of interest to analyze the ADRs produced by atypical antipsychotic drugs, GASS scores, and factors affecting schizophrenic patients for proper management of side effects with a holistic and personalized approach. It is of great importance in the improvement of patient overload and quality of life.

## Materials and Methods:

## Study site and participant:

A prospective observational study of atypical antipsychotic drug-induced adverse drug reactions (ADRs) in schizophrenia patients was conducted in the department of Psychiatry and Pharmacology, KD Medical College, Hospital and Research Center, Mathura, a tertiary care center. Ethical approval was taken before the study from the Institutional Ethics Committee. The total duration of the study was 2 years from Sep 2020 to Aug 2022. The sample size was calculated according to the previous study on the prevalence of ADR among patients receiving antipsychotic drugs by Angadi NB & Mathur C. 2020 [11]. Altogether, a total of 140 patients were included in the study following eligibility criteria of aged between 18 to 70 years and clinically diagnosed with schizophrenia taking atypical antipsychotic drugs. We excluded patients from the study who were not able to provide detailed information regarding medication and adverse effects and those who were on first-generation antipsychotic medication(s) only. Written informed consent was obtained from all participants. Participants were recruited following eligibility criteria. Demographic and clinical characteristics of the patients were noted in the case report form.The details of diagnosed schizophrenic patients from treating physicians were also noted in the specialized case report form. Data on the duration of illness, co-morbidities, medication(s), and adverse effects were collected using a structured questionnaire. Drug treatment exposure included in the study was at least three months during the visit to the psychiatric clinic.

## Data measurement and operational definitions:

ADRs observed during the study were noted according to the definition given by the World Health Organization (WHO) as any noxious and unintended reactions that occur at normal doses used in man, for the prophylaxis, diagnosis, treatment of the disease, and/or modifying physiological functions [12]. Analysis of their probability by using Naranjo's algorithm scale causality assessment method was done and categorized as Definite, Probable, andPossible. A probability scale score > 9 for "Definite reaction", a Score of 5-8 for "Probable reaction" and a score of 1-4 for "Possible reaction" were considered for analysis of the probability of the ADR [13]. Glasgow Antipsychotic Side-effects Scale (GASS) -a modified version, was used to estimate the severity of side effects in each patient. The scale containing the questionnaire was about how patients had been recently. A score of 0-21 was considered "Mild" side effects, 22-42 was considered "Moderate" side effects, and 43-63 was considered "Severe" side effects [14].

## Data analysis:

Data were analyzed for descriptive statistics. A chi-square test was used for the evaluation of the association between patient characteristics (categorical variables and Severity (GASS Score) by using the Statistics Package for Social Science (SPSS) version 25.0 (Chicago SPSS Inc.). P < 0.05 was considered statistically significant.

## Results:

## Demographic and clinical characters of the patient:

A total of 140 patients were included in the study during the study period. The socio-demographic character and clinical character of the patients were presented in Table 1(see PDF) and Table 2(see PDF) respectively. Almost 59 percent of the participants were male. A total of 53 % of patients fall in the age group 40 to 70 years. The educational level of 57 percent of participants was under primary or lower school. 45 participants had co-morbidities including depression, epilepsy, and others (n=25, 15 & 5 respectively). Nearly 66 percent of participants had a total duration of illness of more than 1 year. History of Covid-19 infection taken and it has been reported positive in 39 % of patients at least once during the treatment.

## Drug treatment and adverse effects profile:

The majority (81 %) of patients were on monotherapy for their treatment modality during the study periods. Out of 140 patients, 48 (34 %) received Risperidone while 28.6 % were on olanzapine. A combination of risperidone and trihexyphenidyl was prescribed in 26 (19 %) of the patients (Figure 1 - see PDF). A total of 173 ADRs were reported from 140 participants. Each participant had observed at least one ADR from their drug therapy. Common ADR reported were weight gain (24 %), insomnia (13 %), EPS (12 %), dizziness (12 %), anorexia (10.4 %), and Gastrointestinal upset (8.7 %) (Table 3 -see PDF). Probability assessment of the ADR revealed that 15 % of them were Definite on the Naranjo scale which includes seizure, dizziness, and insomnia. Up on causality assessment offending drug for these reactions was identified and it was olanzapine and risperidone. A majority (55 %) of the ADRs were characterized as Probable while 30 % of ADRs were possible (Figure 2 - see PDF).

## The severity of the side effects (GASS score) and factors affecting:

Analysis of the GASS Score revealed that 55.7 % of participants observed mild side effects from atypical antipsychotic therapy during the study (N=140). Chi-square statistics were applied for the analysis of the association of factors that affect the severity of the GASS score. 41.43 % of females participated in the study out of which 39.7 % of the patient observed to have mild side effects while 60.34 percent patients fall in the group of moderate and severe side effects. Duration of illness of more than 1 year of schizophrenia reported to have a maximum number (54.35) of moderate and severe side effects. A statistically significant result was reported in these groups (Table 4 & Table 5 - see PDF).

## Description:

This study was the one of newest studies that evaluated the association between patient characteristics and severity of side effects in psychiatric disorders who received atypical antipsychotics during the pandemic.In our study sample gender was discovered to be a variable that significantly relates to the outcome of having an adverse effect. A total of 58.57 % of participants were male, but moderate and severe side effects appeared in 60 % of the total female patients. Major patients (81 %) were prescribed monotherapy in the form of risperidone, olanzapine, clozapine, quetiapine, and aripiprazole. Remaining of the patient received combination therapy in the form of risperidone with trihexyphenidyl. 54 % of the poly-therapy group was reported to produce moderate and severe side effects on the GASS score. These finding regarding female patients and combination therapy corroborates with the study of Iversen et al. 2018, which states that women are more likely to experience negative side effects from CNS (central nervous system) acting medications, including psychiatric drugs, mostly because of the drug's pharmacokinetics, which include a higher bioavailability, a slower rate of elimination, and hormonal changes [15]. As the dosing regimen of such drugs is increased or when taken in conjunction with other medications, augmented side effects were documented and are also reported in most of the cases [16]. Given its wide variety of side effects, which include fever, neutropenia, cardiac and pulmonary issues, as well as the potential of clozapine-induced pneumonia,which may contribute to or be mistaken for COVID-19 symptomatology, clozapine should be of great concern for psychiatric patients with COVID-19. An expert group has produced recommendations on the use of clozapine and the treatment of probable adverse effects during the pandemic considering these risks [17]. Only 9 % of the individuals in our sample of acute psychiatric patients were on clozapine, which may have contributed to the low incidence of side effects.In our study, 8.7 % of the total ADR was gastrointestinal upset including constipation, vomiting, and diarrhea. Another typical side effect, particularly for second-generation antipsychotics with a high affinity for cholinergic receptors, is constipation. It is also suggested by the study of Nielsen and Meyer 2012, according to; the patient who was hospitalized for the first episode of psychosis may have experienced constipation because of exposure to anticholinergics used in conjunction with high-potency first-generation antipsychotics, oral administration of second-generation antipsychotics, or due to a less active metabolism while in isolation [18].Different studies have already been conducted to evaluate the quality of life in such types of disorders. They also revealed the patients' quality of life was poor to moderate, and it was inversely associated with the number of unfavorable symptoms, the length of the disease, the total number of previous hospital stays,and the patient's age. Compared to individuals who lived alone or with their families, patients who lived in hostels or group homes had a lower quality of life. Our findings with statistically significant results were found in the group of duration of illness and severity of side effects [19].One of the largest health dangers of our generation is the coronavirus disease pandemic of 2019(COVID-19), which is brought on by the SARS-CoV-2 coronavirus that causes the severe acute respiratory syndrome. The disease'sneuropsychiatric consequences and delirium are present in a sizable percentage of individuals. There have been detected distinct testing results and therapy responses. There is a link between neuropsychiatric symptoms and COVID-19.In this situation, low-potency neuroleptics and alpha-2 adrenergic medications may be especially helpful. The pathophysiology of COVID-19 will need to be further studied to generate more specialized therapy recommendations [20]. In our study, a total of 39 % of patients reported positive once in a treatment period, of which 54.5 % of patients were analyzed as having moderate and severe side effects on GASS score. However, this result was not statistically significant on chi-square estimation. A study of the probability of ADR by atypical antipsychotics in Rajkot, India was carried out. The probability assessment of the ADR by the Naranjo scale revealed by this study was almost similar [21]. The limitation of the study was that we could not include patients on typical antipsychotics or in combination with atypical antipsychotics. Further research may include an association of predictors like biochemical parameters and the study of single nucleotide polymorphism (SNP) in patients with severe side effects.

## Abbreviations: 

ADR: Adverse Drug reaction 

COVID: Corona Virus Disease

GASS: Glasgow Antipsychotic Side-effect Scale 

SARS: Severe Acute Respiratory Syndrome 

 WHO: World Health Organization

## Author contributions:

SMAF & RK contributed to the designing of the study. SP & RK organized the database. SMAF wrote the manuscript. SP & SA worked on data collection and analysis. Author RK helped in literature search and manuscript revision. All authors contributed to manuscript revision, read and approved the submitted version.

## Ethical approval:

This study was approved by the Institutional Ethics Committee, KD Medical College, Hospital & Research Center (Reference Number: KDMCHRC/IEC/2019/10.

## Consent to participate: 

Informed consent to participate in the study was taken from the patient or their legal guardian.

## Consent to publication:

Not applicable Availability of data and materials Not applicable

## Funding:

Not applicable

## References

[1] https://apps.who.int/iris/handle/10665/68782.

[2] Surendiran A (2010). Indian journal of pharmacology..

[3] Routledge PA (2004). Br J Clin Pharmacol..

[4] Stroup TS, Gray N (2018). World Psychiatry..

[5] Wubeshet YS (2019). BMC Psychiatry..

[6] Li M (2016). J Psychopharmacol..

[7] Sahlberg M (2015). J Am Heart Assoc..

[8] Sussman N (2001). J Clin Psychiatry..

[9] Pringsheim T (2017). Can J Psychiatry..

[10] Campion J (2020). Lancet Psychiatry..

[11] https://jnmc.edu/publications/.

[12] Edwards IR, Aronson JK (2000). Lancet..

[13] Naranjo CA (1981). Clin Pharmacol Ther..

[14] Keating D (2017). BMJ Open..

[15] Iversen TSJ (2018). Prog Neuropsychopharmacol Biol Psychiatry..

[16] Siskind D (2020). J Psychiatry Neurosci..

[17] Sönmez Güngör E (2021). Int J Psychiatry Clin Pract..

[18] Nielsen J, Meyer JM (2012). Schizophr Bull..

[19] Browne S (1996). Acta Psychiatr Scand..

[20] Baller EB (2020). Psychosomatics..

[21] Piparva KG (2011). Indian J Psychol Med..

